# An 8-Year-Old Child with Delayed Diagnosis of Netherton Syndrome

**DOI:** 10.1155/2018/9434916

**Published:** 2018-01-30

**Authors:** Alexander K. C. Leung, Benjamin Barankin, Kin Fon Leong

**Affiliations:** ^1^University of Calgary, Calgary, AB, Canada T2M 0H5; ^2^Alberta Children's Hospital, Calgary, AB, Canada T2M 0H5; ^3^Toronto Dermatology Centre, Toronto, ON, Canada M3H 5Y8; ^4^Pediatric Institute, Kuala Lumpur General Hospital, Kuala Lumpur, Malaysia

## Abstract

We report an 8-year-old boy with Netherton syndrome who was misdiagnosed and treated as severe atopic dermatitis. The diagnosis of Netherton syndrome was not made until the child was 8 years of age. We discuss the pitfalls in the diagnosis and alert physicians to the proper and early diagnosis of this syndrome. The child was treated with a low dose (0.25 mg/kg) of oral acitretin and a topical moisturizer with marked improvement of his skin and pruritus in 2 months. At 6-month follow-up, the skin was almost clear of erythema and scaling, and the hair was longer and stronger. The dose of acitretin was reduced to 0.12 mg/kg for another 6 months and then discontinued.

## 1. Introduction

Netherton syndrome is an autosomal recessive disorder of cornification characterized by the triad of congenital ichthyosiform erythroderma/ichthyosis linearis circumflexa, trichorrhexis invaginata (bamboo hair), and an atopic diathesis. The condition was first described in 1958 by Earl Netherton who reported a girl with an erythematous scaly dermatitis and bamboo-like nodes in her sparse fragile hair [1]. The disease now bears his name.

We describe a Malay boy with Netherton syndrome in whom the diagnosis was not made till he was 8 years old. We discuss the pitfalls in the diagnosis and alert physicians to the proper and early diagnosis of this syndrome.

## 2. Case Report

An 8-year-old Malay boy presented with generalized migratory serpiginous pruritic scaly patches and sparse hair. He was born at term to a para 1 gravida 2 25-year-old mother at 39 weeks gestation following an uncomplicated pregnancy and normal vaginal delivery. Parents were nonconsanguineous. He was noted to have erythroderma and desquamation involving most of the body shortly after birth. His physician diagnosed him as having severe atopic dermatitis and treated him with intensive topical emollients, corticosteroids, and a calcineurin inhibitor. The eruption waxed and waned, but never completely cleared. There was no history of recurrent infections of the skin, upper respiratory tract, or gastrointestinal tract. The developmental milestones were normal. The child had atopic dermatitis and asthma and was allergic to eggs and nuts. He had no family history or hair anomalies or similar skin disorders. His 45-year-old father had allergic rhinitis and his 10-year-old sister had atopic dermatitis and asthma.

**Figure 1:**
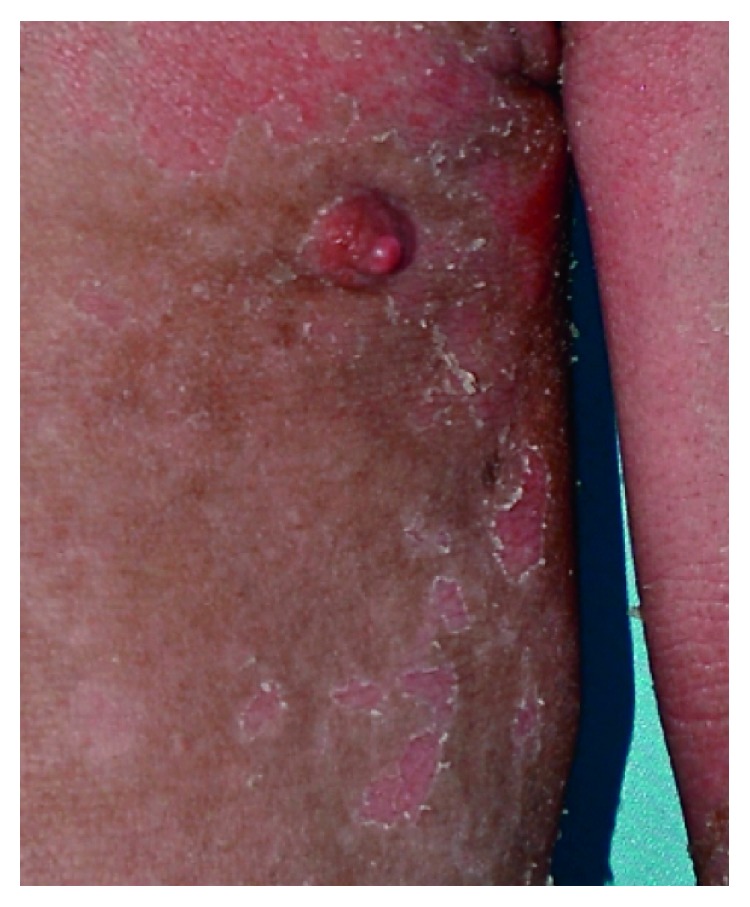
Serpiginous, erythematous plaques with double-edged peripheral scale.

**Figure 2:**
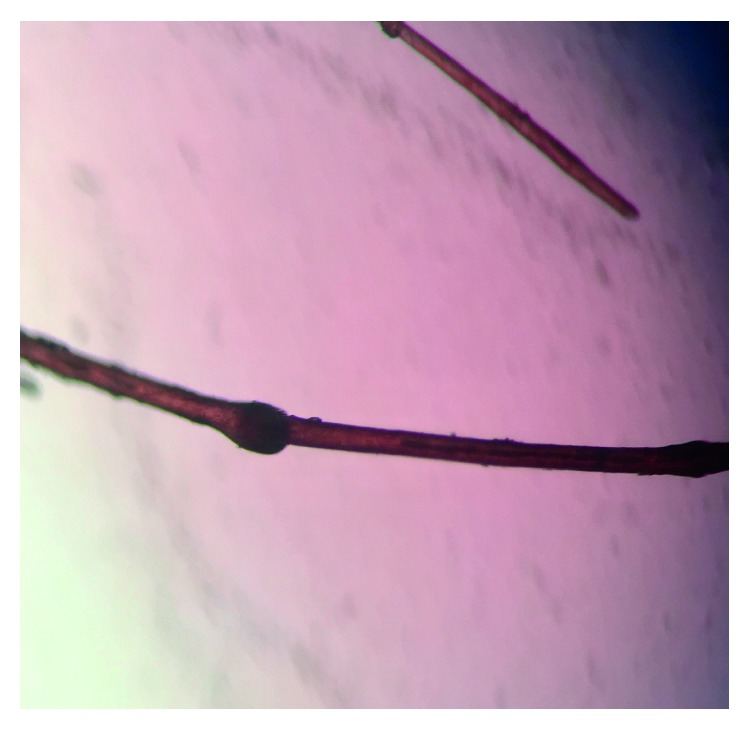
Trichoscopic examination of the child's scalp hair showing trichorrhexis invaginata (bamboo hair).

On examination, his height was 124 cm (25th percentile) and weight was 21.5 kg (10th percentile). There were widespread serpiginous, erythematous patches with double-edged peripheral scaling typical of ichthyosis linearis circumflexa on the body (Figure 1). The skin was dry with flexural lichenification. The scalp hair was short, brittle, and sparse. The eyelashes and eyebrows were sparse. Trichoscopic examination of the scalp hair showed trichorrhexis invaginata (bamboo hair) (Figure 2). The rest of the physical examination was normal.

Laboratory investigations showed peripheral eosinophilia (absolute eosinophil count of 25,000/mm^3^) and a serum IgE level of 5,500 IU/mL (normal < 100 IU/mL). Radioallergosorbent tests (RASTs) using specific IgE antibodies were positive to milk, egg, nut, dust mite, and pollen. Serum levels of IgG, IgA, and IgM were normal.

**Figure 3:**
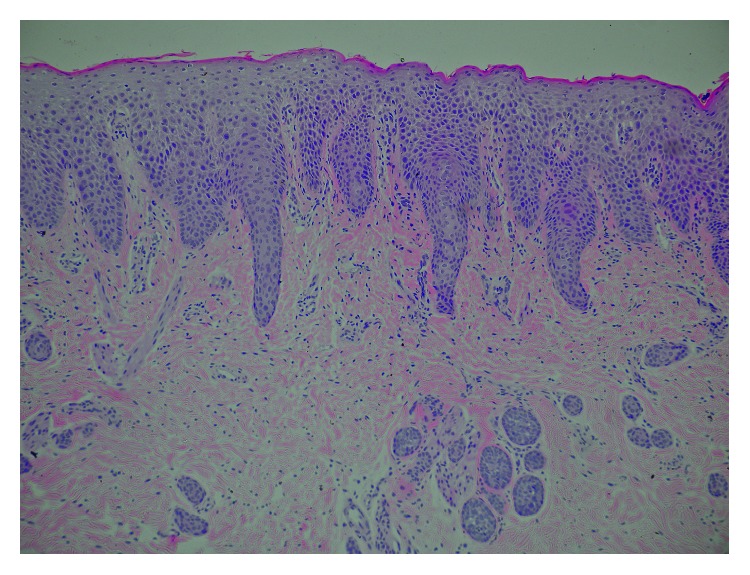
A skin biopsy showing “irregular” acanthosis, parakeratosis, and psoriasiform epidermal hyperplasia.

A skin biopsy showed “irregular” acanthosis, parakeratosis, and psoriasiform epidermal hyperplasia (Figure 3). Immunohistochemistry using specific antibodies showed complete absence of lymphoepithelial Kazal-type-related inhibitor (LEKTI) in the biopsy specimen.

This patient was treated with oral acitretin 0.25 mg/kg and a topical moisturizer with marked improvement of the skin lesions and pruritus in 2 months. Treatment was well tolerated with no significant adverse effects. At 6-month follow-up, the skin was almost clear of erythema and scaling, and the hair was longer and stronger. The dose of acitretin was reduced to 0.12 mg/kg for another 6 months and then discontinued. Genetic counseling was also offered.

## 3. Discussion

Netherton syndrome is caused by germline mutations in the serine protease inhibitor of Kazal type 5 (*SPINK5*) gene located on chromosome 5q31-32 [2]. The gene encodes a serine protein kinase known as lymphoepithelial Kazal-type inhibitor (LEKTI), expressed in mucosal and epithelial surface [2–4]. These mutations result in an unopposed activity of epidermal proteases leading to premature desquamation of the stratum corneum and impairment of the skin barrier. It is estimated that the condition affects 1 in 100,000 to 200,000 live births [3, 5].

Clinically, Netherton syndrome is characterized by the triad of congenital ichthyosiform erythroderma/ichthyosis linearis circumflexa, trichorrhexis invaginata (“bamboo hair” or “ball and socket” hair shaft deformity), and an atopic diathesis [5, 6]. Congenital ichthyosiform erythroderma is often present at birth or shortly thereafter and manifests as generalized erythroderma and desquamation. Some infants are born with a collodion membrane. Over time, the erythroderma evolves into ichthyosis linearis circumflexa characterized by migratory, serpiginous, erythematous, patches/plaques with double-edged scales at the periphery [7]. Pruritus is a constant feature [7]. Ichthyosis linearis circumflexa waxes and wanes throughout the patient's life [5]. Lichenification of antecubital and popliteal fossae is common [7].

The hair is typically lusterless, dry, sparse, short, beaded, brittle, and easily broken [8–10]. Trichorrhexis invaginata of hair and eyebrows, due to invagination of the distal portion of the hair shaft into the proximal portion, is pathognomonic for Netherton syndrome [10, 11]. When hair breaks at the point of invagination, the appearance simulates a “golf tee” or “matchstick” [7–9]. Some patients may have trichorrhexis nodosa and pili torti. Hair shaft abnormalities usually do not develop until later in infancy or early childhood and are best visualized on trichoscopy or trichogram [5].

Atopic manifestations include atopic dermatitis, asthma, allergic rhinitis, urticaria, angioedema, anaphylactic reactions to food, blood hypereosinophilia, and elevated serum IgE [4, 5, 10].

Recurrent infections, especially bacterial infections of the skin, are common and occur in at least 30% of affected patients [4]. In one study, recurrent infections occurred in almost all affected patients [4]. Other inconsistent features that may be present include heat intolerance, mental retardation, growth retardation, hypoalbuminemia, enteropathy, and exocrine pancreatic insufficiency [2, 4, 12].

In the presence of characteristic cutaneous findings (ichthyosiform erythroderma/ichthyosis linearis circumflexa), trichorrhexis invaginata, atopic diathesis, and positive family history, the diagnosis is straightforward. However, when the features are atypical or absent, the diagnosis may be delayed or missed.

Netherton syndrome can be misdiagnosed as atopic dermatitis due to the presence of eczematous lesions in Netherton syndrome and personal/family history of atopy, as is illustrated in the present case. This is especially so when specific features such as ichthyosis linearis circumflexa; sparse, short, and brittle hair; and trichorrhexis invaginata are not evident. In general, trichorrhexis invaginata and ichthyosis linearis circumflexa do not become evident until after one year of age and two years of age, respectively [13]. In addition, serum IgE levels are only mildly elevated in the early stage of Netherton syndrome and therefore cannot be used to differentiate the two conditions.

In the present case, although the child had erythroderma and desquamation shortly after birth, the diagnosis of Netherton was overlooked because there was no family history of the syndrome and the hair was not affected in early infancy and the fact that hair may be sparse in healthy infants in the first few months of life. As well, the nature and severity of the skin lesions are variable between and within patients, rendering the diagnosis difficult. Also, ichthyosis linearis circumflexa might not always be present and can alternate with erythematosquamous plaques [14].

Typically, the diagnosis is delayed until the appearance of trichorrhexis invaginata which is pathognomonic. Therefore, hair examination by trichoscopy or trichogram should be carried out early so that the diagnosis would not have been missed or delayed. It is not unusual for hundreds of hairs to be examined before a hair with trichorrhexis invaginata can be found [11]. The hair shaft abnormality is best visualized in eyebrow hair [8, 9, 11]. The diagnosis can be confirmed, if necessary, by identification of a germline *SPINK5* mutation by DNA sequencing [3]. However, the cost of performing DNA sequencing analysis limits its use in diagnosis [9].

Currently, there is no known cure or satisfactory treatment for Netherton syndrome. Treatment modalities such as topical corticosteroids, topical calcineurin inhibitors, topical retinoids, narrowband ultraviolet B phototherapy, psoralen and ultraviolet irradiation, and oral acitretin have been used with varying success [2, 15, 16]. Nevet et al. treated a 6-month-old girl with Netherton syndrome with oral acitretin 1 mg/kg and topical humectants which led to marked improvement of the erythema and scaling [2]. Lazaridou et al. treated a 14-year-old male with oral acitretin 0.2 mg/kg which resulted in gradual remission of the skin lesions [15]. On the other hand, other investigators found that treatment with oral acitretin aggravated the skin condition [16]. The present report showed that a low dose (0.25 mg/kg) of oral acitretin was effective in the treatment of the cutaneous manifestations of this disease. Intravenous immunoglobulin and anti-TNF-*α* are new therapeutic options in those with severe illness [4, 6, 7]. It is hoped that further understanding of the basic pathophysiology of integumentary changes will lead to more effective therapeutic modalities.

## 4. Conclusion

In the presence of characteristic cutaneous findings, trichorrhexis invaginata, atopic diathesis, and positive family history, the diagnosis of Netherton syndrome is straightforward. However, when the features are atypical or absent, the diagnosis may be delayed or missed. Typically, the diagnosis is delayed until the appearance of trichorrhexis invaginata which is pathognomonic. Hair examination should be carried out early so that the diagnosis is neither missed nor delayed.
